# PDGF/PDGFR effects in osteosarcoma and the “add-on” strategy

**DOI:** 10.1186/s13569-018-0102-1

**Published:** 2018-08-02

**Authors:** Jie Xu, Lu Xie, Wei Guo

**Affiliations:** Peking University People’s Hospital, Beijing, 100044 China

**Keywords:** PDGF, PDGFR, Osteosarcoma, Add-on therapy

## Abstract

New treatment options for advanced osteosarcoma have remained limited. The platelet-derived growth factor (PDGF)/platelet-derived growth factor receptor (PDGFR) pathway plays an important role in the development and metastasis of osteosarcoma, via either direct autocrine stimulation of tumor cells, or paracrine stimulation on tumor stromal cells. It promotes angiogenesis to overcome hypoxia in the tumor microenvironment, and modulates tumor interstitial fluid pressure to control the influx and efflux of other agents. Targeting the PDGF/PDGFR pathway is a promising therapeutic method to overcome drug resistance and improve patients’ outcome in osteosarcoma. Further evidence is needed to define the detailed mechanism. Results from clinical trials using PDGF/PDGFR inhibitor as a single agent were disappointing, both in osteosarcoma and soft tissue sarcoma. However, when combined with other agents, named as “add-on” strategy, a synergistic antitumor effect has been confirmed in soft tissue sarcoma, and should be attempted in osteosarcoma.

## Background

Osteosarcoma is the most common primary bone malignancy in adolescents and young adults, with an incidence rate of 4.4 cases per 1 million each year in people aged 0–24 years [1]. Current multidisciplinary treatments have led to a dramatic improvement in prognosis for patients with localized osteosarcoma; long-term survival rates of less than 20% improved to 65–70% [2]. Unfortunately, with metastatic disease, the rate of long-term survival is greatly reduced to 20–30% [3, 4]. Although several clinical trials have been conducted, and new drugs such as sorafenib and everolimus have been tested in metastatic osteosarcoma, the overall outcome has not significantly improved over the past few decades [5–7]. The event-free survival for refractory or relapsed osteosarcoma is as low as 12% at 4 months [7].

Platelet-derived growth factors (PDGFs) and their receptors (PDGFRs) are major players in oncogenesis and drug resistance, and are attractive oncologic targets in cancer [8]. PDGFs/PDGFRs are frequently expressed in various tumors, and their expression levels correlate with tumor growth, invasiveness, drug resistance, and poor clinical outcomes [9]. The PDGF pathway is also a key signal for tumor stromal cells recruitment such as cancer-associated fibroblasts [10], which play an important role in the maintenance of tumor microenvironment. The exact mechanism leading to the development and metastasis of osteosarcoma is not fully understood. The PDGF receptor does not appear to be as central to proliferation for osteosarcoma cultures as Bcr/Abl is for chronic myelogenous leukemia. Although PDGF is known to stimulate proliferation and differentiation of osteoblasts and osteoclasts [11], its role in osteosarcoma, specifically PDGF ligands and their receptors, is poorly understood. Whether treatment with PDGF/PDGF receptor antagonists will be beneficial for osteosarcoma is the subject of ongoing studies. In this article, we summarize the preclinical and clinical studies of the PDGF pathway in osteosarcoma, and discuss the mechanism and future directions.

## Overview of PDGF signaling

### Activation and function of PDGF/PDGFR pathway

The platelet-derived growth factor (PDGF) signaling network consists of four ligands, PDGF-A, PDGF-B, PDGF-C, and PDGF-D, and two receptors, PDGFR-α and PDGFR-β. All PDGFs function as disulfide-linked homodimers, but only PDGF-A and -B can form functional heterodimers. Before binding to the protein tyrosine kinase receptors PDGFR-α and PDGFR-β, the four PDGF ligands are inactive in their monomeric forms. The receptor isoforms dimerize upon binding, leading to three possible receptor combinations, namely -AA, -BB, and -AB, causing the subsequent activation of kinase. Kinase activation is visualized as tyrosine phosphorylation of the receptor molecules, which occurs between the dimerized receptor molecules (transphosphorylation). The receptor molecules then undergo conformational changes that allow a basal kinase activity to phosphorylate a critical tyrosine residue, thereby “unlocking” the kinase, leading to full enzymatic activity directed toward other tyrosine residues in the receptor molecules, as well as other substrates for the kinase [12, 13].

More than 10 different molecules bind selectively to phosphorylated residues in the PDGF receptors, including the Src family, SHP-2 tyrosine phosphatase, phospholipase C-γ (PLC-γ), and the GTPase activating protein (GAP) for Ras. Furthermore, the receptors bind and activate signal transducers and activators of transcription (STATs). Finally, some of the receptors binding molecules lack intrinsic enzymatic activities, but can form complexes with other signaling molecules. For example, the regulatory subunit p85 of the PI3K forms a complex with the p110 catalytic subunit, and Grb2 activates Ras and the Erk MAP-kinase pathway by binding with SOS1. In addition, the PDGF receptors bind other adaptors (e.g., Shc, Nck, Crk, and GAB) and mediate interactions with numerous different downstream signaling molecules [14, 15]. The activation of these signaling pathways leads to cell survival, proliferation, angiogenesis, and cell migration.

PDGFs and PDGFRs are expressed by a large variety of normal human tissues and organs. PDGFs are major mitogens for many cell types of mesenchymal or neuro-ectodermal in origin. PDGFs have chemo-attractant properties and are involved in erythropoiesis, wound healing, bone formation, and angiogenesis. Much evidence suggests involvement in the normal development of important organs such as kidney, brain, and cardiovascular and respiratory systems [16]. During normal development, cell proliferation significantly increases as a consequence of PDGF overexpression and decreases in PDGF null mutants.

### PDGF/PDGFR pathway in tumor development and metastasis

A complex interplay between cancer cells, endothelial cells, and other stromal cells occurs in tumor angiogenesis. The PDGF/PDGFR pathway plays an important role in the development and metastasis of tumors in at least three different ways: (1) direct autocrine stimulation of tumor cells [17]; (2) paracrine stimulation of tumor stromal cells [12] and promotion of angiogenesis to overcome hypoxia in the tumor microenvironment [18]; (3) modulation of tumor interstitial fluid pressure (IFP) to control the influx and efflux of agents [19].

The mode of action of PDGF and PDGFR involvement in tumor development and progression is mainly through direct autocrine stimulation of tumor cell growth and cell autonomy, whereas in normal tissues paracrine stimulation is predominant [20]. Direct blockade of autocrine stimulation using inhibitors of PDGFR in cell lines and xenograft models show positive results in tumors such as lung cancer [21], hepatic cancer [22], gastrointestinal stromal tumor (GIST) [23], dermatofibrosarcoma protuberans [24], and osteosarcoma, which expressed PDGFR-α on the cell surface [25]. The levels of phosphorylation of both PDGFRs and the downstream signaling proteins such as protein kinase B (AKT) and extracellular regulated kinase (ERK) are downregulated by PDGFR antibodies or small molecular tyrosine kinase inhibitors (TKIs) [25].

In addition to direct autocrine stimulation, the PDGF/PDGFR pathway exerts paracrine stimulation on tumor stromal cells. PDGF promotes the formation of a rich stromal compartment, characterized by deposition of extracellular matrix components and blood vessel formation [26]. Experimental models showed that VEGF-null cells require PDGFR to recruit fibroblasts in tumor stroma [27]. Cancer-associated fibroblasts (CAFs), which secrete several active factors to stimulate angiogenesis and tumor invasion, constitute a functionally important component of tumor stroma in many types of cancer [28].

Clinical data indicate that carcinomas with desmoplastic stroma, consisting of fibroblast cells and extracellular matrix, are associated with a poor prognosis [29]. An experimental model of glioma revealed that PDGF-B enhances angiogenesis by stimulating VEGF expression in tumor-associated endothelial cells and by recruiting pericytes [30]. Several other preclinical models demonstrated that using antibodies or TKIs to disrupt the paracrine signaling with PDGFR can significantly reduce tumor growth by inhibiting tumor cell growth, recruitment of fibroblasts, and angiogenesis in xenograft tumor models or genetic mouse models of cancer [31], such as melanoma [26], cervical cancer [32], and colon cancer [33].

Targeting the PDGF/PDGFR pathway can regulate the influx and efflux of agents and thus modify the uptake of drugs. Previous data showed that connective tissue cells control interstitial fluid pressure (IFP) by exerting tension on the collagen/microfibrillar network via collagen binding integrin a_2_b_1_ [34, 35]. Experiments using transgenic mice carrying PDGFR-β receptor mutations suggest a function for PDGF signaling through PI3K in IFP homeostasis by modulating the tension between cells and extracellular matrix structures [35]. Previous studies reported that the inhibition of PDGFR reduced interstitial hypertension and increased transcapillary transport in tumors. An increased tumor uptake of the tracer compound ^51^Cr-EDTA and paclitaxel, after inhibition of PDGF receptor using TKIs in tumor stroma, was previously demonstrated [18, 19, 36].

An increased tumor uptake of cytotoxic drugs has therefore been proposed as the mechanism and further promotes combination treatment with PDGF antagonists and chemotherapy, which is called an “add-on” strategy. “Add-on” strategy has been used in a variety of tumors with different partners, such as fludarabine phosphate (F-AMP) in GIST [37], doxorubicin in breast cancer [38], and fucoxanthin in chronic myeloid leukemia [39]. Because of the mild adverse effect of PDGF antagonists, most combinations are safe and well-tolerated.

## PDGF/PDGFR pathway in osteosarcoma

### Expression of PDGFs/PDGFRs in osteosarcoma

The expression of PDGF/PDGF receptors in osteosarcoma has been tested in immunohistochemical (IHC) studies of specimens and various cell lines. A significant heterogeneity has been found in this pathway because the proportion of positive staining varies widely from study to study (4–90%). However, the association of higher expression of PDGF with higher proliferation and poorer prognosis seems universally accepted. One of the early immunohistochemical studies demonstrated that of 37 cases of osteosarcoma, 38% showed PDGF and PDGFR expression, with 11 (30%) cases having expression of both, and correlation of PDGF-positive tumors with higher proliferation (MIB-1 index) compared with PDGF-negative tumors [40]. Another immunohistochemical study also demonstrated that 23 osteosarcoma specimens each expressed both PDGF-A and PDGF-a receptor with 52 and 43% staining strongly positive (> 25% of the cells stained), respectively [41]. A study with 57 osteosarcoma samples showed that high levels of PDGF-AA expression were associated with a significantly lower 5-year disease-free survival compared with low-level expression of PDGF-AA (21.22% vs. 42.72%). PDGF-a receptor expression, although exhibiting a similar trend, failed to achieve significance [42]. A flow cytometry study of patient-derived osteosarcoma cell lines showed high expression of PDGFR-β in the co-expression with insulin receptors [43].

In a clinical trial for imatinib and osteosarcoma, a frequent expression of PDGF-AA (80.4%) and PDGF-a receptor (79.6%) and their correlation with inferior event-free survival (P < 0.05) was reported. PDGF-BB and PDGF-b-receptor expressions were also frequent (75.4 and 86%, respectively); however, statistically significant inferior event-free survival was not demonstrated (P = 0.15) [44]. On the contrary, in a larger study of 100 patients, 96 showed PDGFRA expression ranging from 4 to 90%. Overall and disease-free survival analysis did not reveal any differences between osteosarcoma patients, according to the level of PDGFRA expression [45]. Another study showed more than 50% positive expression of PDGFs/PDGFRs but no relationship with patients’ outcome [46].

### Targeting PDGF/PDGFR pathway in osteosarcoma

By definition, autocrine growth is suggested to occur in cells expressing both ligand and its cognate receptor, and osteosarcomas meet these criteria [17]. Co-expression of PDGF and PDGFRs has been confirmed in osteosarcoma cell lines, as well as in human osteosarcoma samples, as mentioned above. Recently, antagonists specific for PDGF/PDGFR have been developed and used to investigate the role of PDGFR stimulation in various diseases [14, 15, 47]. Typically, there are two kinds of inhibitors, (1) the antibody or other binders can target the receptors and prevent their activation or promote their degradation specifically [48, 49], and (2) several tyrosine kinase inhibitors of PDGF receptor kinases (TKIs), including imatinib, sunitinib, sorafenib, and pazopanib, which are not specific and are multi-targeted [47, 50–52] (Table 1). For most TKIs studied in osteosarcoma clinical trials, anti-angiogenesis was considered a more dominant mechanism compared with anti-PDGF effect. The only exception was imatinib.

**Table 1:** The IC_50_ values and targets of the approved kinase inhibitors against PDGFRs

Kinase name	Imatinib	Dasatinib	Nilotinib	Sorafenib	Sunitinib	Pazopanib
IC_50_ values						
PDGFR-α [49]	2.5	2.6	2.4	1.0	13	20
PDGFR-α [T674I] [50]	8500		4100	100	19	170
PDGFR-α [V561D] [50]	9.1	2.9	15	5.6	14	21
PDGFR-β [53]	68	1.1	60	34	5.7	42
Primary targets [53, 54]	Abl, PDGFR, Kit	Abl, Src	Kit, Abl, PDGFR	Raf, VEGFR, PDGFR, Kit, Flt3	PDGFR, VEGFR, Kit, Flt3	VEGFR, PDGFR, Kit
Secondary targets [53, 54]	Raf	PDGFR, Kit				FGFR

Primary targets: The targets that are inhibited at the lowest concentrations (regardless of absolute concentrations)

Secondary targets: The targets that are inhibited by about tenfold higher inhibitor concentrations

#### Imatinib

Imatinib mesylate (Gleevec, STI571, Novartis Pharmaceuticals) was the first TKI developed as an ATP competitive inhibitor of ABL tyrosine kinase that has been highly effective in chronic myelomonocytic leukemia [53, 54]. Imatinib also inhibits other tyrosine kinase receptors except for ABL, including PDGFR and c-kit, and was approved for treatment of c-kit positive gastrointestinal stromal tumors (GIST) and dermatofibrosarcoma protuberans [24]. Given the activation of PDGF/PDGFR pathway, several in vitro and in vivo experiments have been performed with osteosarcomas.

Because PDGF acts as a potent mitogen in several osteosarcoma cell lines [55, 56] and patient-derived cell cultures [44], the inhibition of the PDGF pathway results in a reduction of cell proliferation due to caspase-dependent apoptosis [44, 56, 57], an arrest of the cell cycle, and an inhibition of cell migration. Imatinib exhibits a dose-dependent anti-proliferative effect in all cell lines studied [56]. To further define the inhibition effect of PDGF pathway, the expression of downstream signaling protein has been tested. Greater than 50% inhibition of PDGFR phosphorylation and its downstream signaling molecules such as AKT and ERK is observed after treatment with imatinib. An in vivo study shows a redundancy in growth factor loops in osteosarcoma. Implantation of TE-85 and MG-63 cells into the tibia of nude mice revealed that lower levels of PDGF were sufficient to satisfy the PDGFRs [17]. However, unlike the promising results from in vitro studies, the effect of imatinib alone in animal models was limited [17], which disallows use of a PDGF inhibitor as a single drug.

Less efficacy as a single drug was also demonstrated in two open labeled, single arm phase 2 clinical trials, namely NCT00031915 and NCT0003066. In the NCT00031915 trial, imatinib was used in patients with one of 10 different subtypes of advanced sarcoma. Patients were treated with imatinib 300 mg bid. In 27 patients with osteosarcoma, no complete response (CR) or partial response (PR) but only 5 stable disease (SDs) were recorded [58]. In another phase 2 trial by the Children’s Oncology Group (COG), NCT0003066, imatinib was administered daily for 28 day courses at a dose of 440 mg/m^2^/day. In 70 eligible patients, only one PR was seen among 24 patients with Ewing sarcoma. No objective responses were confirmed in osteosarcomas [59]. The failure of these clinical trials suggests that use of imatinib alone in advanced osteosarcoma is not appropriate.

Although imatinib alone had no antitumor effect in mice harboring OS tumors or in clinical trials, using imatinib as an add-on therapy is more promising. Synergistic antiproliferative effects of imatinib and doxorubicin has been noticed in PDGFR-expressing osteosarcoma cells both in vitro and in vivo [60]. Combined antiproliferative activity with cisplatin has also been demonstrated in experimental Ewing sarcoma [61]. The synergistic effect can be partly explained by the reversion of multidrug-resistance (MDR) related transporters [62]. The on-going clinical trials using imatinib as add-on therapy could give us more information (Table 2).

**Table 2:** Ongoing study using “add-on” strategy with PDGF/PDGFR inhibitors in solid tumors

Regimen	Mechanism of partner	Diagnosis	Phase	Study design	Start date	Recruitment	ClinicalTrials.gov Identifier
Imatinib+							
Pemetrexed	Cytotoxic drugs	Pleural mesothelioma	2	Single group, open label	11/2014	Active, not recruiting	NCT02303899
Temzolomide	Cytotoxic drugs	Melanoma	1/2	Single group, open label	01/2003	Active, not recruiting	NCT00667953
Regoragenib	Multi-targeted TKI	GIST	2	Randomized, open label	02/2015	Recruiting	NCT02365441
BYL719	PI3K inhibitor	GIST	1	Single group, open label	02/2013	Active, not recruiting	NCT01735968
Pembrolizumab	Check point inhibitor	Melanoma	1/2	Single group, open label	11/2016	Recruiting	NCT02812693
Letrozole	Antihormonal drug	Breast cancer	2	Single group, open label	10/2003	Active, not recruiting	NCT00338728
Binimetinib	MEK inhibitor	GIST	1b/2	Single group, open label	11/2013	Recruiting	NCT01991379
BGJ398	FGFR inhibitor	GIST	1b/2	Single group, open label	10/2014	Active, not recruiting	NCT02257541
Ipilimumab	Check point inhibitor	Cancer	1	Single group, open label	02/2013	Recruiting	NCT01738139
Olaratumab+							
Paclitaxel/carboplatin	Cytotoxic drugs	NSCLC	2	Randomized, open label	01/2010	Active, not recruiting	NCT00918203
Nab-paclitaxel/gemcitabine	Cytotoxic drugs	Pancreatic cancer	1b/2	Randomized, double-blind	01/2017	Recruiting	NCT03086369
ADM	Cytotoxic drugs	STS	3	Randomized, double-blind	09/2015	Active, not recruiting	NCT02451943
Standard chemos	Cytotoxic drugs	Pediatric cancer	1	Non-randomized, open label	08/2016	Recruiting	NCT02677116
ADM	Cytotoxic drugs	STS	2	Non-randomized, open label	02/2016	Recruiting	NCT02584309
Gemcitabine/ADM	Cytotoxic drugs	STS	1b/2	Randomized, double-blind	03/2016	Recruiting	NCT02659020
Trabectedin/ADM	Cytotoxic drugs	Leiomyosarcoma	1	Non-randomized, open label	Not yet	Not yet recruiting	NCT03437070
ADM	Cytotoxic drugs	STS	1	Single group, open label	10/2016	Active, not recruiting	NCT02783599
ADM	Cytotoxic drugs	Cancer	1	Non-randomized, open label	03/2016	Active, not recruiting	NCT02377752
ADM/ifosfamide	Cytotoxic drugs	STS	1	Single group, open label	10/2017	Recruiting	NCT03283696
Pembrolizumab	Check point inhibitor	STS	1	Non-randomized, open label	07/2017	Recruiting	NCT03126591

*PI3K* phosphatidylinositol 3-kinase, *STS* soft tissue sarcoma, *ADM* adriamycin

As a target of multiple TKIs, the activity of imatinib can also be explained by other mechanisms, such as the inhibition of transforming growth factor(TGF) and its cross-talk with c-Myc, which was upregulated in MG63 cell lines. Imatinib can also inhibit cell proliferation by blocking TGF and c-Myc [63].

#### Olaratumab

Olaratumab is a human immunoglobulin G subclass 1 mAb with selective, high affinity binding to the extracellular domain of PDGFR-α, disrupting receptor ligand interactions with resulting downregulation of downstream signal transduction [64]. Preclinical studies of olaratumab alone [65] or in combination with doxorubicin have demonstrated antitumor activity in human sarcoma xenograft models, and several other kinds of cancers [66, 67].

A recent evaluation of the effect of olaratumab in osteosarcoma [25] concluded that in vitro olaratumab treatment of osteosarcoma and rhabdoid tumor cell lines reduced proliferation and inhibited invasion driven by individual platelet-derived growth factors (PDGFs) or serum. Furthermore, olaratumab delayed primary tumor growth in mouse models of pediatric osteosarcoma and malignant rhabdoid tumor, and this activity was enhanced by combination with either doxorubicin or cisplatin [25].

Similar to imatinib, the use of olaratumab alone is unsatisfactory in clinical trials, whereas its combination with other drugs seems more promising. Two phase 1 studies evaluated the effect of olaratumab as a single agent in patients with advanced sarcomas [68, 69]. In both studies, olaratumab was well tolerated and without dose-limiting toxicities. However, no objective radiological response was recorded in either study. The best response of SD was shown in 12 (63%) and 7 (44%) patients, respectively. When combined with doxorubicin, a recent phase 2 study (JGDG) on soft tissue sarcoma showed a surprising effect of olaratumab as an “add-on” agent [70]. In the pivotal trial, the combination of olaratumab with doxorubicin reduced the risk of death by 48% compared with doxorubicin alone (HR, 0.52; 95% CI 0.34–0.79, P < 0.05). Median overall survival in the intent-to-treat population (n = 129) was improved by 11.8 months. The median overall survival was 26.5 months with the combination versus 14.7 months with doxorubicin alone. Olaratumab has been also tested in other malignancies, such as GIST [23] and lung cancer [23]. Unlike the success of add-on therapy in soft tissue sarcoma, a 131-patient phase 2 trial in treatment-naïve adult patients with advanced non-small cell lung cancer (NSCLC) failed to show any advantage when olaratumab was added to the classical carboplatin/paclitaxel regimen, with an increasing risk of neutropenia, thrombocytopenia, and fatigue in the experimental arm [21] (Table 3).

**Table 3:** Published phase 1/2 studies of olaratumab

Author	Phase	N	Diagnosis	ORR	CBR	PFS	OS
Chiorean [69]	1	19	Solid tumors	0	NA	NA	NA
Doi [68]	1	16	Solid tumors	0	NA	NA	NA
Tap [70]	1b	15	Soft tissue sarcoma	NA	NA	NA	NA
Tap [70]	2	129	Soft tissue sarcoma	Olaratumab+ ADM 18.2% ADM alone 11.9%	NA	6.6 months 4.1 months	26.5 14.7
Wagner [25]	2	21	GIST	PDGFR-a mutation(+) PDGFR-a mutation(−)	50.0 14.3	32.1 weeks 6.1 weeks	NR 24.9 weeks
Gerber [23]	2	131	NSCLC	olaratumab+ P/C P/C	NA	4.4 months 4.4 months	11.8 months 11.5 months

*N* number; *ORR* objective response rate, =CR+PR; *CBR* clinical benefit rate, =CR+PR+SD at 12 weeks; *PFS* progression free survival; *OS* overall survival; *NA* not available; *ADM* adriamycin; *NR* not reached; *P/C* paclitaxel/carboplatin

## Development and possible mechanisms for add-on strategy

### Promising strategy in add-on therapy

Given the unsatisfactory result of clinical trials using imatinib as a single drug, it is clear that targeting PDGF/PDGFR alone would not be sufficient to control the growth of osteosarcoma, even in tumor cells that express PDGF/PDGFR highly. The PDGF signaling pathway does not appear to be the main driver of osteosarcoma cells, especially in the presence of other growth-promoting factors such as VEGF. Osteosarcomas have rich vascularity and many growth signals, which may also influence the unfavorable results obtained in clinical trials. The application of PDGF/PDGFR pathway targeted therapy would therefore be expected to be effective only if added to other therapy.

Data from phase I and phase 2 trials showed that the adverse effect of most PDGF/PDGFR inhibitors was mild, which allow the opportunity of delivering “add-on” therapy. Adding olaratumab to ADM, the first-line chemo agent in soft tissue sarcoma, has achieved great success [70]. The strategy of “add-on” therapy is used in many ongoing studies in different types of solid tumors (Table 3). The addition of PDGF/PDGFR inhibitors to standard chemotherapy of osteosarcoma should be attempted in the future to overcome drug-resistance and to improve patients’ outcome.

### Possible mechanisms for add-on strategy

Two possible mechanisms can explain the results of combination drug therapy using PDGF/PDGFR inhibitors.

The first hypothesis is overcoming tissue hypoxia in tumor microenvironment, a common phenomenon in sarcomas. Tissue hypoxia occurs where there is an imbalance between oxygen supply and consumption. Hypoxia occurs in solid tumors as a result of an inadequate supply of oxygen, due to exponential cellular proliferation and an inefficient vascular supply. It is an adverse prognostic indicator in cancer as it is associated with resistance to therapy [71, 72]. The crosstalk between hypoxia inducible factor (HIF-11α) and PDGF has been investigated in several cell lines [73, 74]. The use of PDGF inhibitors could improve hypoxia in tumor tissue, therefore leaving them more sensitive to other drugs [8].

The second hypothesis is based on a regulation of tumor interstitial fluid pressure (IFP) brought by PDGF inhibition, which could eventually increase tumor uptake of other agents [19, 75]. Lowering of tumor interstitial hypertension, which acts as a barrier for tumor transvascular transport, has been proposed as a general strategy to enhance tumor uptake and therapeutic effects of anticancer drugs. As mentioned above, the effects of PDGF antagonists on chemo responses as a mediator of tumor hypertension have been tested in various types of malignancies [18, 19, 34–36]. Although treatment with only PDG Fantagonists had no effect on tumor growth, uptake of classical cytotoxic drugs in tumors was increased by treatment with PDGF antagonists.

## Future research in PDGF/PDGFR pathway

### PDGF/PDGFR pathway in stromal cells

The development and progression of cancer depend on the microenvironment. Cancer cells themselves cannot explain growth and formation of the primary or metastasis, and a combination of proliferating tumor cells and their partners, including cancer stem cells, immune cells, mesenchymal stromal cells, and/or CAFs all contribute to the tumor bulk [76–78]. Studies of other types of malignant tumors strongly suggest that PDGF isoforms secreted by cancer cells affect the stromal microenvironment by inducing fibroblast proliferation, migration, infiltration, myofibroblastic conversion, and overproduction of ECM components, and finally promoting tumorigenesis indirectly in a paracrine manner [14, 26].

Compared to tumor cells, stromal cells show a higher expression of PDGFRs [79, 80]. According to the conclusion of the JGDG study, no difference was found in objective response rate or survival considering the expression level of PDGFR, indicating that the indirect manner of targeting tumor microenvironment may be more important in therapy with PDGF/PDGFR inhibitors. More studies of PDGF/PDGFR pathways in stromal cells of osteosarcoma are needed to explore further the mechanism and activity, including but not limited to the associations between the PDGFR status of stromal cells and the extent of malignancy, the ability of metastasis, response to treatment, and survival conditions.

### PDGF/PDGFR inhibitors in drug-resistant cells

Although synergistic antiproliferative effects of PDGF/PDGFR inhibitors, such as imatinib and olaratumab, and classical cytotoxic agents (i.e., AMD, cisplatin) were observed in both in vitro and in vivo experiments, the effects of these inhibitors on drug-resistant cells have not been explored. Intratumoral and intertumoral heterogeneity was found in drug-resistant osteosarcoma cell lines [81] and in samples from refractory patients [82, 83]. Relapsed osteosarcoma is characterized by complex signaling and drug resistance pathways. However, the abnormal efflux of cytotoxic agents demonstrated a common mechanism of multiple drug resistance [84, 85]. The effect of PDGF inhibitors to upregulate drug uptake in tumor cells and to overcome tissue hypoxia has been described above, but the mechanism of reversal of drug is not well understood. More research is needed with drug-resistant cells to confirm this action.

## Conclusion

New treatment options for advanced osteosarcoma remain limited. The PDGF/PDGFR pathway plays an important role in the development and metastasis of osteosarcoma, via both tumor cells and stromal cells. Targeting the PDGF/PDGFR pathway is a promising therapeutic method to overcome drug-resistance and to improve patients’ outcome. The “add-on” strategy, adding PDGF/PDGFR inhibitors to other existing agents, should be attempted in the future.
